# Improved hematopoietic differentiation of human pluripotent stem cells via estrogen receptor signaling pathway

**DOI:** 10.1186/s13578-016-0111-9

**Published:** 2016-08-30

**Authors:** Hye-Ryun Kim, Jong-Hee Lee, Hye-Ryeon Heo, Se-Ran Yang, Kwon-Soo Ha, Won Sun Park, Eun-Taek Han, Haengseok Song, Seok-Ho Hong

**Affiliations:** 1 Department of Biomedical Science, College of Life Science, CHA University, 689 Sampyeong-dong, Bundang-gu, Seongnam, 463-400 Republic of Korea; 2 Stem Cell and Cancer Research Institute, McMaster University, Hamilton, ON L8N 3Z5 Canada; 3 Department of Internal Medicine, School of Medicine, Kangwon National University, Kangwondaehakgil 1, Chuncheon, Gangwon 200-701 Republic of Korea; 4 Department of Thoracic and Cardiovascular Surgery, School of Medicine, Kangwon National University, Chuncheon, Republic of Korea; 5 Department of Molecular and Cellular Biochemistry, School of Medicine, Kangwon National University, Chuncheon, Republic of Korea; 6 Department of Physiology, School of Medicine, Kangwon National University, Chuncheon, Republic of Korea; 7 Department of Medical Environmental Biology and Tropical Medicine, School of Medicine, Kangwon National University, Chuncheon, Republic of Korea; 8 Stem Cell Institute, Kangwon National University, Chuncheon, Republic of Korea

**Keywords:** Estrogen, Human pluripotent stem cells, Hematopoiesis, Erythrocytes

## Abstract

**Background:**

Aside from its importance in reproduction, estrogen (E2) is known to regulate the proliferation and differentiation of hematopoietic stem cells in rodents. However, the regulatory role of E2 in human hematopoietic system has not been investigated. The purpose of this study is to investigate the effect of E2 on hematopoietic differentiation using human pluripotent stem cells (hPSCs).

**Results:**

E2 improved hematopoietic differentiation of hPSCs via estrogen receptor alpha (ER-α)-dependent pathway. During hematopoietic differentiation of hPSCs, ER-α is persistently maintained and hematopoietic phenotypes (CD34 and CD45) were exclusively detected in ER-α positive cells. Interestingly, continuous E2 signaling is required to promote hematopoietic output from hPSCs. Supplementation of E2 or an ER-α selective agonist significantly increased the number of hemangioblasts and hematopoietic progenitors, and subsequent erythropoiesis, whereas ER-β selective agonist did not. Furthermore, ICI 182,780 (ER antagonist) completely abrogated the E2-induced hematopoietic augmentation. Not only from hPSCs but also from human umbilical cord bloods, does E2 signaling potentiate hematopoietic development, suggesting universal function of E2 on hematopoiesis.

**Conclusions:**

Our study identifies E2 as positive regulator of human hematopoiesis and suggests that endocrine factors such as E2 influence the behavior of hematopoietic stem cells in various physiological conditions.

**Electronic supplementary material:**

The online version of this article (doi:10.1186/s13578-016-0111-9) contains supplementary material, which is available to authorized users.

## Background

Estrogen (E2), a primary steroid hormone, plays a crucial role in the development, maturation, and functions of male and female reproductive organs [1, 2]. E2 acts on target cells by binding nuclear receptors, of which two nuclear receptors have been identified, estrogen receptor (ER)-α and ER-β [3]. The E2-ER complex binds to specific sequences of DNA and then modulates transcription of its target genes, called genomic action of E2. E2 can also mediate rapid signaling independent of genomic pathway. This rapid signaling can be mediated by various intracellular second messengers, which confer to the ability of rapid transmission of E2 signaling in target cells [4].

Aside from its importance in reproduction, a growing body of evidence indicates that E2 is involved in regulating the proliferation and differentiation of multipotent and pluripotent stem cells [5–7]. A recent study unveiled an unexpected function of E2 in promoting cycling of hematopoietic stem cells (HSCs) and multipotent progenitors (MPPs) and their differentiation into megakaryocyte-erythroid progenitors (MEPs) [8]. While the administration of pharmacologic agonists and antagonists of ER seems to modulate HSC proliferation in vivo, to date, comparatively less is known about the regulatory role of E2 in early hematopoietic development in humans. Furthermore, as expected, it is very difficult to understand such coordinated events of E2 on human hematopoiesis in vivo context. Human pluripotent stem cells (hPSCs) have provided promising opportunities to understand the fundamental processes of human cell fate decisions in the context of tissue regeneration and human diseases [9]. The process of hematopoietic development in vivo is tightly controlled and regulated by the distinct intrinsic and extrinsic signaling pathways and in vitro hematopoiesis of hPSCs mimicks these signaling cascades active during embryonic development [10–12]. Consistent with the importance of Notch, Wnt and Hedgehog (Hh) signalings during early embryonic hematopoiesis, recent evidence has shown that activation of these signaling pathways is crucial for both the emergence of hemogenic cells and the subsequent hematopoietic specification from hPSCs [10, 13]. Thus, we utilized hPSCs as a robust in vitro system to investigate the function of E2 during hematopoietic cell fate decision.

In this study, we identify a unique role of E2 in the regulation of hematopoiesis from hPSCs. E2-ER-α signaling enriches hemangioblasts, hematopoietic progenitors and subsequent erythrocytes during hematopoietic differentiation programs in humans.

## Results

### ER-alpha is persistently maintained during hematopoietic differentiation

**Fig. 1:**
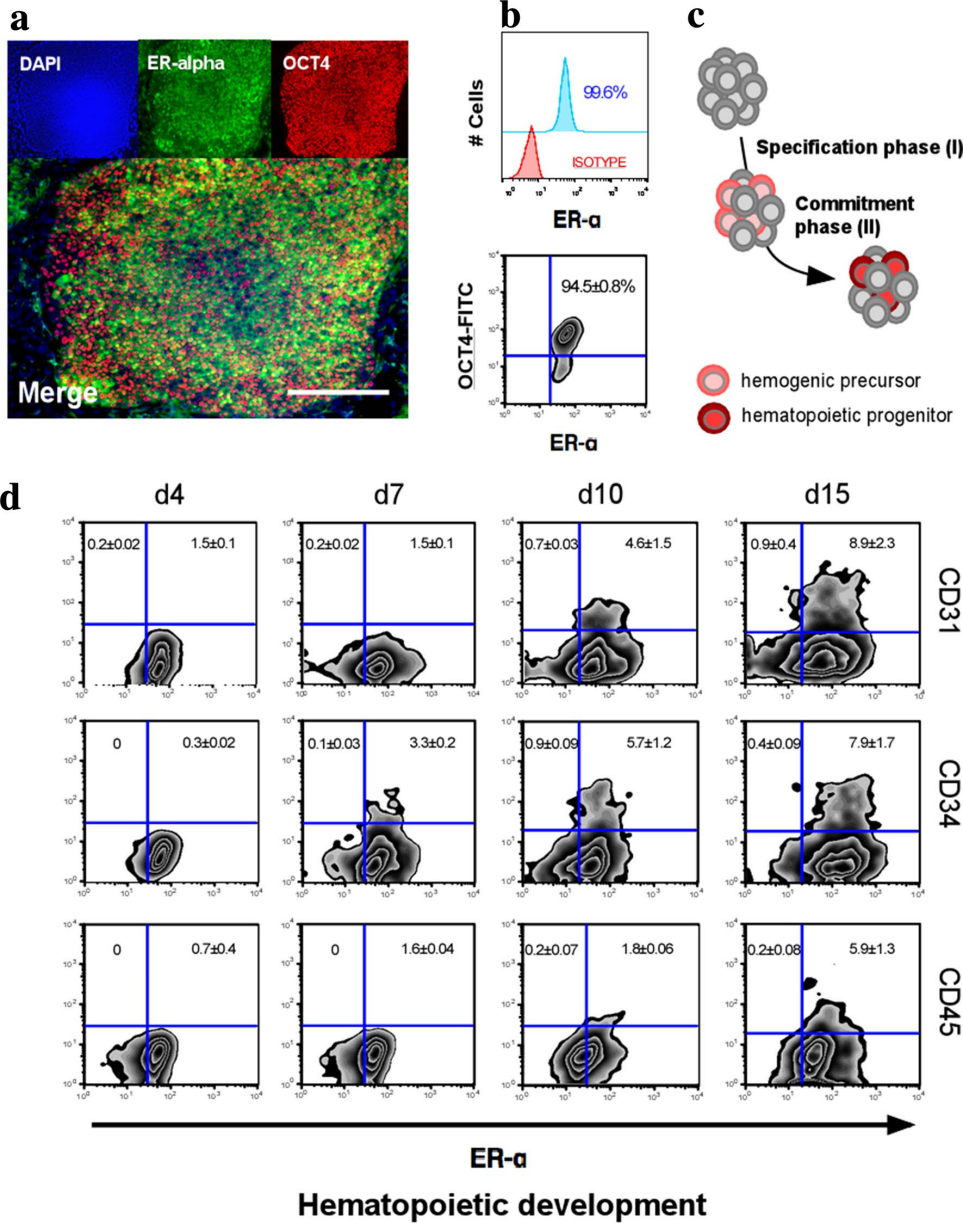
Temporal expression of ER-α and hematopoietic markers during hiPSC-derived hematopoietic development. **a** Immunocytochemistry staining for ER-α (*green*) costained with OCT4 (*red*) in feeder-free hiPSC cultures. Nuclei were counterstained with DAPI (*blue*). *Scale bar* 100 μm. **b** Frequencies of ER-α and OCT4 in undifferentiated hiPSC cultures by flow cytometry. **c** Schematic diagram of hematopoietic development from hPSC: hemogenic specification phase (phase I) and hematopoietic commitment phase (phase II). **d** Temporal expression patterns of ER-α and hematopoietic markers during hiPSC-derived hematopoietic development

To understand potential roles of E2 during the development of human hematopoietic systems, we first examined the expression of ER in hPSCs. Previously, we demonstrated that steroid receptors, such as ER-α, ER-β, glucocorticoid receptor, and progesterone receptor, are expressed in undifferentiated hPSCs and EBs [6]. Consistent with the previous report, ER-α is highly expressed in undifferentiated colony of hPSCs (Fig. 1a). Interestingly, ER-α was detected even in spontaneously differentiated OCT4 negative cells as well (Fig. 1b). To demonstrate the role of E2 signaling in hematopoietic differentiation, we used a hEB differentiation model from hPSCs. As previously established [14], hierarchical stages of hematopoiesis can be characterized into two phases of hEB development, emergence of bipotential hemogenic precursors (CD45_neg_PFV) and development of hematopoietic progenitors (CD34^+^CD45^+^) and mature blood (CD34^−^CD45^+^) cells (Fig. 1c). Whereas the number of ER-α positive cells was progressively down-regulated during in vitro hematopoietic development of hPSCs (Additional file 1: Figure S1), expression of ER-α is highly maintained in cells within hematopoietic lineage. Hemogenic precursors (CD45_neg_PFV) and hematopoietic cells (CD34^+^CD45^+^ and CD34^−^CD45^+^) were exclusively included in ER-α positive cells (Fig. 1d; Additional file 2: Table S1). These data collectively suggest that E2 may have a novel function on the regulation of hematopoietic lineage specification in humans.

### Continuous E2 signaling is required to promote hematopoietic output from hPSCs

**Fig. 2:**
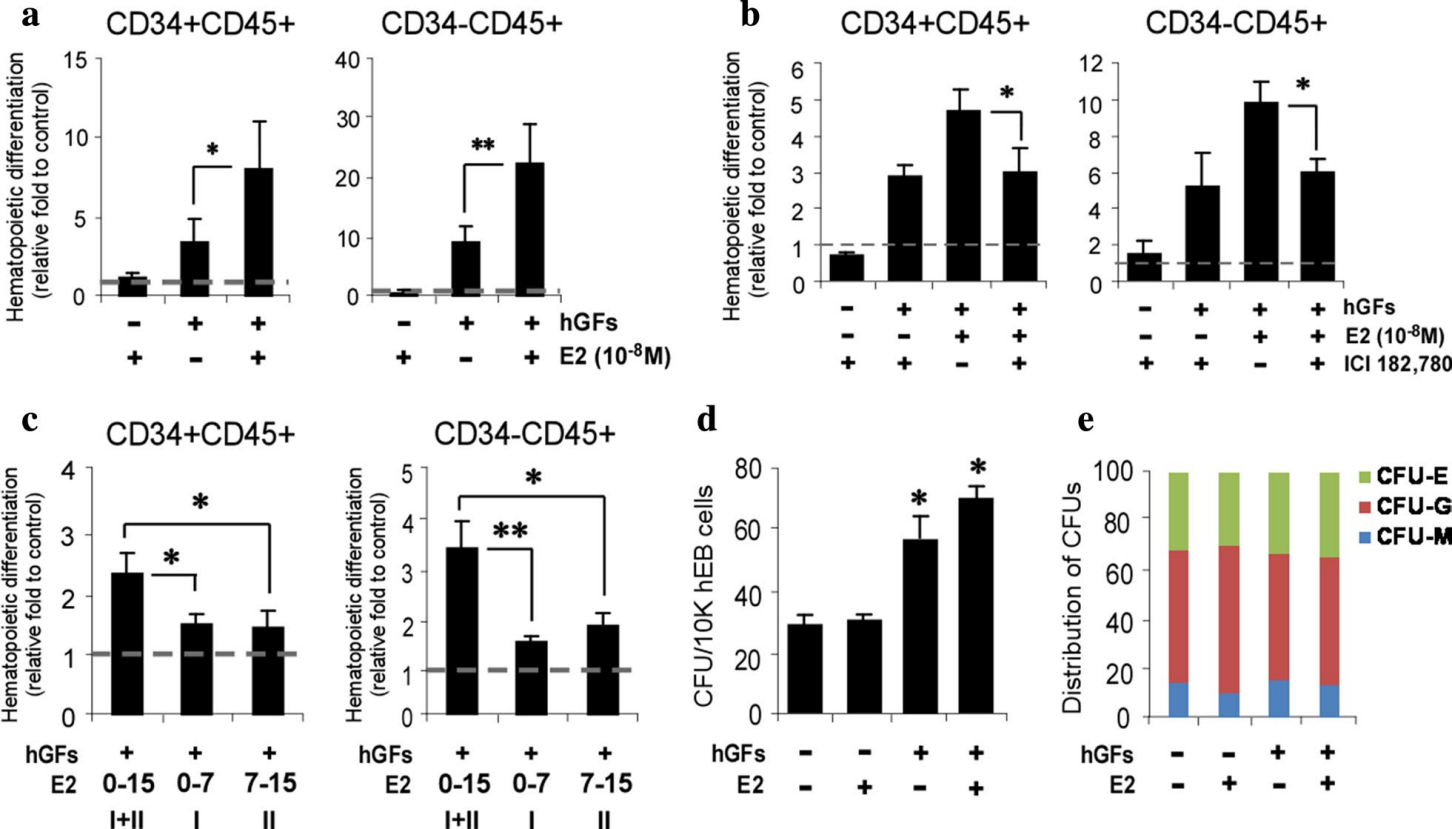
Effect of E2 in hiPSC-derived hematopoiesis. **a**, **b** Effects of E2 on the production efficiency of hematopoietic progenitors (CD34^+^CD45^+^) and mature hematopoietic cells (CD34^−^CD45^+^) from hiPSC-derived EBs at day 15. EBs were cultured in hematopoietic induction medium alone or supplemented with different combination of E2, hGFs and ICI 182,780. **c** Assessment of hematopoietic output by restricting the period of E2 exposure during EB differentiation to phase I (I) and phase II (II) alone compared to differentiation during both phase I and II (I+II). **d** Assessment of hematopoietic progenitor capacity of EBs differentiated under various treatments for 15 days. **e** Distribution of CFU types (CFU-E, erythroid; CFU-M, macrophage; and CFU-G, granulocytes). All results are mean ± SD. *p < 0.05, **p < 0.01

To examine functional role of E2 during hematopoiesis, E2 at various concentrations was added to culture media during hematopoietic differentiation of hEBs in the presence or absence of optimized human growth factors (hGFs) [15]. E2 at 10^−8^ M and 10^−7^ M provided synergistic effects with hGFs on hematopoietic differentiation (Additional file 3: Figure S2). E2 at 10^−8^ M concentration was used for following experiments since it is physiologically more relevant [16, 17]. As suggested by our previous report [15], hGFs exhibited a dramatic increase of hematopoietic progenitors (CD34^+^CD45^+^) and mature blood (CD34^−^CD45^+^) cells. In this context, E2 further promoted output of hematopoietic progenitors and matured blood cells approximately twofold more (Fig. 2a). However, E2 treatment without hGFs did not have any effects on it, suggesting cooperative actions of E2 with hGFs to improve hematopoietic differentiation of hPSCs. We then asked if E2 differentially regulates hematopoietic differentiation in male (DF-699T.B) and female (IISH3i-CB6) hPSCs. There were no significant differences in the frequency of hematopoietic progenitors and mature blood cells between male and female hPSCs (Additional file 4: Figure S3A, B). To validate that enhanced blood differentiation by E2 is mediated via nuclear ERs, ICI 182,780, an ER antagonist, was added during hematopoietic differentiation of hPSCs. ICI 182,780 significantly interfered with actions of E2 on hematopoietic development in both primitive and matured blood cells (Fig. 2b). Furthermore, suppression of ER-α expression in undifferentiated hPSC cultures using siRNA reduced the output of hematopoietic differentiation (Additional file 4: Figure S3C, D). These results strongly indicate that E2-induced enrichment of hematopoietic cells is ER dependent during hematopoiesis of hPSCs irrespective of gender of hPSCs.

Hematopoietic differentiation program can be divided into two phases, hemogenic specification (days 0–7) and then hematopoietic commitment (days 7–15). To investigate temporal effects of E2 signaling during hematopoietic differentiation, hEBs were treated with E2 for hemogenic specification (days 0–7) phase, hematopoietic commitment (days 7–15) phase or both phases (days 0–15). In cases that E2 was temporally provided, both primitive and matured hematopoietic output was significantly reduced as compared with those of E2 treatment for both phases. These results strongly suggest that continuous activation of E2 signaling is required for E2-dependent increase of hematopoietic differentiation (Fig. 2c). We next examined whether E2 could promote functional multi-lineage capacity of hematopoietic progenitors as well using the in vitro CFU assay. While supplementation of hGFs significantly increased total number of CFUs produced by progenitor cells, E2 did not show any synergistic effects on this event (Fig. 2d). Furthermore, the distribution of CFU types was not affected by any treatments (Fig. 2e), suggesting that E2 signaling does not have critical influence on the functional capacity of hematopoietic progenitors. Taken together, these results demonstrated that E2-ER signaling pathway facilitates hPSC-derived hematopoietic programming, but not multi-lineage potential of hematopoietic progenitors.

### E2 significantly increases the number of hemangioblasts and erythroid colonies via ER-α-dependent pathway

**Fig. 3:**
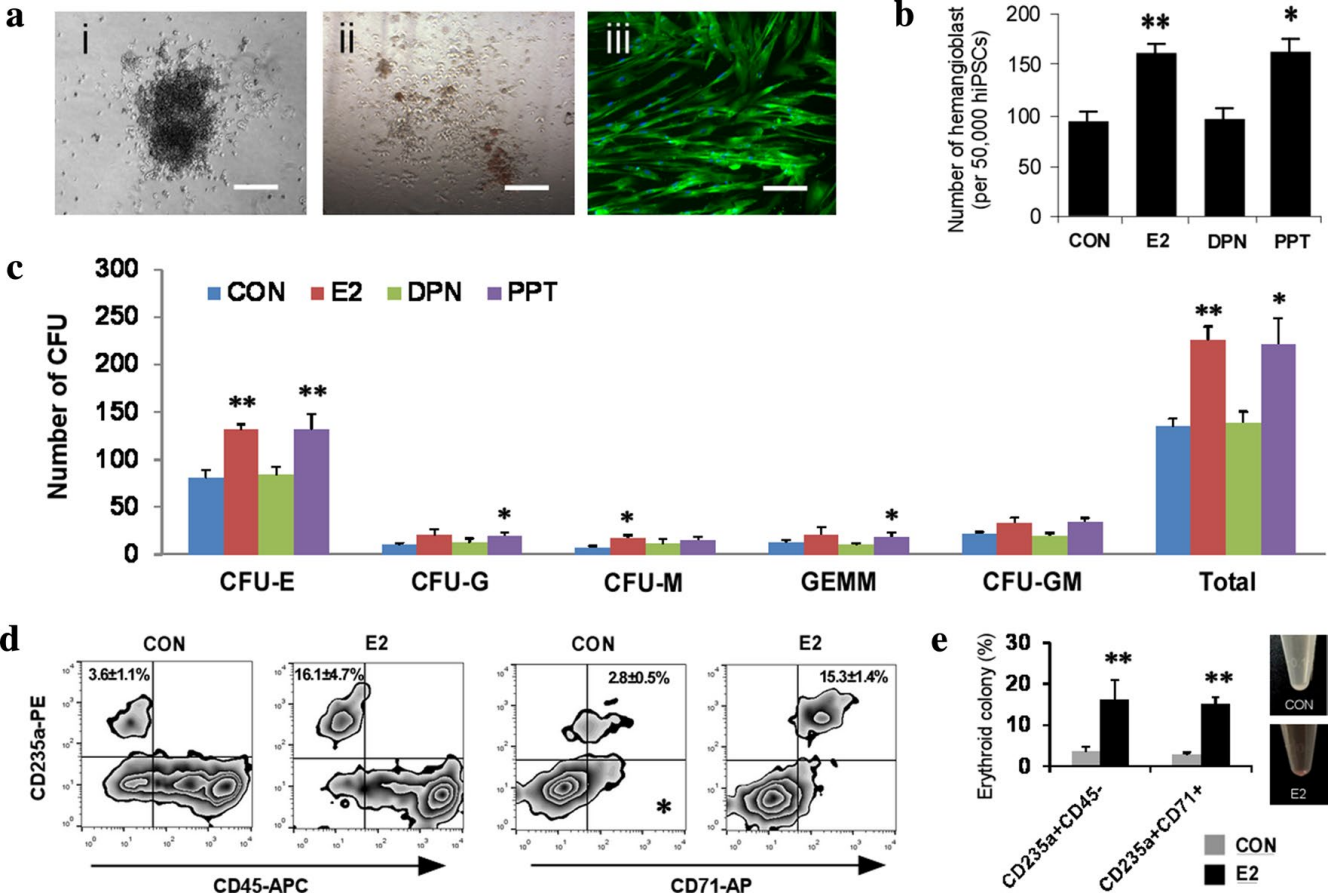
Increased number of hemangioblasts and erythroid colony during iPSC hematopoiesis is dependent on ER-α signaling. **a** Representative image of a hemangioblast derived from hiPSCs (i) Multiple types of hematopoietic CFU (ii) and endothelial cells (iii, stained with CD144) derived from hiPSC-hemangioblasts. **b**, **c** Effects of E2, DPN and PPT on the generation of hemangioblasts from hiPSCs and their hematopoietic progenitor capacity. **d**, **e** Effects of E2 on the output of CFU-E from hiPSC-derived hemangioblasts were analyzed by flow cytometry. All results are mean ± SD. *p < 0.05, **p < 0.01

To understand whether enhanced hematopoietic differentiation by E2 is specified from the hemogenic cells, retaining both hematopoietic and endothelial potential [18, 19], we assessed effects of E2 on the ability of hPSCs to generate developmentally intermediate hemangioblasts. As shown in Fig. 3a, hPSCs could produce hemangioblasts that further differentiated into both endothelial and hematopoietic lineages. Interestingly, E2 produced larger number of hemangioblast colonies from hPSCs (Fig. 3b), suggesting that E2 signaling is involved in fate decision process during early hematopoiesis. Furthermore, in case that same number of hemangioblast cells is provided, E2 generated significantly more CFUs from them (Fig. 3c), suggesting that ER signaling potentiates not only hemogenic specification, but also subsequent hematopoietic lineage development of hPSCs. So far, two nuclear receptors have been identified, ER-α and ER-β in mediating E2 signal pathway (Hall et al. 2001). To further investigate which receptor works for E2-mediated hematopoietic development, PPT (100 nM) and DPN (100 nM), selective agonists of ER-α and ER-β, were used to produce hemangioblasts, respectively. The activation of ER-α with PPT, but not ER-β with DPN, significantly enhanced the generation of hemangioblasts (Fig. 3b) and hematopoietic commitment (Fig. 3c), similar extents with E2, suggesting that a novel function of E2 during hematopoiesis of hPSCs is exclusively dependent on ER-α. Given the recent evidence that E2 preferentially give rise to CFU-E from hematopoietic progenitors, we further examined erythrocyte generation by applying an optimized protocol to generate functional erythrocytes from human embryonic stem cells (Kennedy et al. 2007). The expression of high levels of Glycophorin A (CD235a) with the lack of CD45 (CD235a^+^CD45^−^) and co-expression of CD235a and CD71 (CD235a^+^CD71^+^) on their surface confirmed erythrocytic features of differentiated cells (Fig. 3d). E2 notably increased the frequency of erythrocyte markers, hence it suggests that activation of E2 signaling pathway enhances capacity of erythropoietic differentiation capacity from hPSCs (Fig. 3e). Taken together, these results demonstrate that facilitated hematopoietic differentiation capacity of E2 is specified at the early hemangioblastic development from the pluripotent state.

### E2 promotes the expansion of hematopoietic progenitors derived from hUCBs

**Fig. 4:**
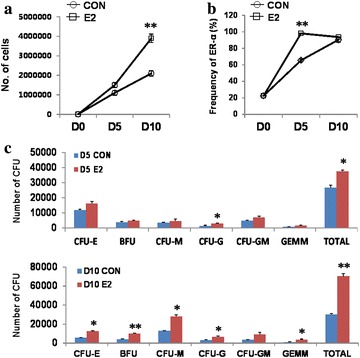
Effect of E2 in the proliferation and differentiation of hematopoietic progenitors derived from hUCB. **a** Hematopoietic progenitors (CD34^+^) were harvested from hUCB using MACS and exposed to E2 for 5 and 10 days. Effects of E2 on the proliferation of hUCB-derived hematopoietic progenitors. **b** Frequencies of ER-α were measured by flow cytometry. **c** Assessment of the hematopoietic multi-lineage potential of hUCB-derived hematopoietic progenitors treated with E2 for 5 and 10 days. All results are mean ± SD. *p < 0.05, **p < 0.01

Since we newly identified E2-ER-α signaling enable to potentiate hematopoietic development of hPSCs, next we examined whether E2 has similar function in hematopoietic progenitors (CD34^+^) from human umbilical cord bloods (hUCBs). Fraction of hematopoietic progenitors was purified from hUCBs and cultured in the presence of E2. First, we examined whether E2 can promote proliferation of hematopoietic progenitors by counting cell numbers for 10 days culture. E2 significantly increased the number of blood cells (approximately twofold increase at day 10) (Fig. 4a). Although no significant differences in cell number are detected at day 5, fraction of ER positive cells is highly maintained in the presence of E2 (Fig. 4b). Taken together, these results demonstrate that E2 augmented the proliferation of hematopoietic progenitors by maintaining ER positive cells. To further understand E2 actions on the multi-lineage potential of hematopoietic progenitors, we performed CFU assays from cells cultured for 5 or 10 days in the presence of E2. E2 significantly enhanced the CFU output even at 5 days culture, suggesting that E2 signaling promotes functional capacity of hematopoietic progenitors in addition to proliferation (Fig. 4c). Increased number of diverse types of CFUs at day 10 further suggested that most hematopoietic lineages are influenced by E2 signaling.

## Discussion

HSCs is maintained by various intrinsic factors and environmental cues in vivo or in vitro [10–12]. Therefore, understanding molecular mechanisms by which these regulatory factors maintain HSCs is required to instruct successful proliferation and maintenance of HSCs for clinical applications. Our current study identifies a critical novel function of E2 during early human hematopoiesis and the expansion of hematopoietic progenitors. Although this report is the first to reveal a role for E2 signaling in early human hematopoietic specification, similar mechanisms have been shown to regulate hematopoiesis in other species. Significant reduction of HSCs in E2-deficiency rat by ovariectomy has been reported and this hematopoietic dysfunction in the bone marrow (BM) may be due to altered levels of hGFs such as SCF and IL-3 [20]. In mice, E2 increases numbers of functional short-term HSCs (ST-HSCs) with reconstitutive potential in the vascular niche by enhancing S-phase entry [21]. A recent study demonstrated the differential expression of ER-α and ER-β between long-term (LT) repopulating HSCs (LT-HSCs) and committed progenitors in the mouse BM [22]. It also showed that ER-α deficiency led to the reduction of LT- and SH-HSCs, but did not affect the number of MPPs, suggesting distinct role of E2 pathway in subpopulations of mouse HSCs. In addition, E2-ER-α signaling yielded elevated HSC division and erythropoiesis especially in female mice [8]. Although these results in other species provided practical implications for E2 in human HSCs, it has not been achieved to date partly due to the lack of appropriate human models. In this aspect, we provide a model to investigate early human hematopoiesis in vitro with hPSCs and demonstrate positive effect of E2 on this developmental process.

In vitro differentiation of hPSCs provided valuable surrogate for understanding the cellular and molecular mechanisms that govern developmental process of the human in vivo. Based on our present study using hPSCs, we propose a beneficial role of E2 signaling in promoting intrinsic hematopoietic potential of hPSCs and further demonstrated that the activation of ER-α enhances generation of hemangioblasts and erythrocytes. Nakada et al. also showed that activation of ER signaling with PPT but not with DPN significantly increased HSC division as well as erythropoiesis in the BM and spleen [8]. These data indicate that E2 effects on HSCs and erythropoiesis are mediated primarily via ER-α and functionally conserved between mice and humans. While this study clearly demonstrates that continuous activation of ER signaling improves hematopoietic differentiation in humans, downstream signaling pathways that regulate these processes still remain elusive. Recently, it was reported that EGR1, a member of EGR family of transcription factors, controls both the proliferation and mobilization of HSCs in mouse BM [23]. Several groups including us recently showed that E2-ER signaling pathway transiently induces EGR1 to orchestrate a second wave of gene expression in the uterus [24–26]. We found that c-KIT, a transmembrane receptor tyrosine kinase for stem cell factor, is one of EGR1 direct target genes whose transcription is directly regulated by EGR1 in the uterus (data not shown). It is interesting to note that c-KIT is an important cell surface marker sued to identify hematopoietic stem/progenitor cells in the BM and c-KIT + hPSCs are lineage-biased toward hematopoietic cell fate [27, 28]. In this regard, it is possible that E2 induces immediate early response genes such as EGR1 to produce local factors critical for hematopoiesis in human BM and hematopoietic development of hPSCs. Further studies are warranted to characterize underlying molecular mechanisms by which E2 controls hematopoietic differentiation in humans.

Consistent with increased hematopoiesis in hPSCs, E2 pathway appeared to potentiate functional activity of hematopoietic progenitors of hUCBs. The small number of hematopoietic stem/progenitor cells in UCBs limits their widespread use for transplantation and gene therapy [29, 30]. Thus, efficient and continuous ex vivo expansion of purified hematopoietic stem/progenitors without loss of repopulation capacity has become a priority to meet the clinical requirements for allogeneic transplantation [31]. In this respect, our data suggest that expansion of hematopoietic progenitors with multi-lineage potential of UCBs by E2 signaling enables UCBs to become a prioritized source for future clinical applications.

## Conclusions

The present study demonstrates that E2 improved hematopoietic differentiation of hPSCs via ER-α-dependent pathway. Taken together, the present finding expands our understanding of actions of endocrine factors on human hematopoietic development, which may provide new possible potential candidate for HSC transplantation. It is definitely critical to evaluate in vivo repopulation capability of hematopoietic stem/progenitors expanded in the presence of E2 for clinical cell transplantation therapies.

## Methods

### Maintenance of hiPSCs

All experiments were carried out with the hiPSC lines DF-699T.B (WiCell) and IISH3i-CB6 (WiCell). Undifferentiated hiPSC lines were maintained on Matrigel (BD Biosciences)-coated six-well tissue culture plates in mTeSR1 serum-free medium (STEMCELL Technologies). The cells were passaged at a 1:4 split ratio every 5 days by mechanical dissociation and the medium was replaced daily.

### Hematopoietic differentiation and knockdown of ER-α transcript using siRNA

For hematopoietic differentiation of hiPSCs through embryoid body (EB) formation, confluent undifferentiated hiPSCs were scraped off and transferred to Ultra-Low attachment plate (Corning). The hiPSC clumps were incubated overnight in EB differentiation medium [Knock-out-Dulbecco’s modified Eagle’s medium (KO-DMEM) supplemented with 20 % fetal bovine serum (FBS, Hyclone), 1 mM l-glutamine, 0.1 mM β-mercaptoethanol, and 1 % non-essential amino acids]. At the next day, the medium was changed with the same EB differentiation medium supplemented with hematopoietic growth factors (hGFs): 25 ng/ml bone morphogenetic protein-4 (BMP-4), 300 ng/ml stem cell factor (SCF), 10 ng/ml interleukin-3 (IL-3), 10 ng/ml IL-6, 50 ng/ml granulocyte colony stimulating factor (G-CSF), and 300 ng/ml Flt-3L. Thereafter, the medium was changed every 3 days. All hGFs were purchased from R&D systems. To investigate the effect of E2 (Sigma) on hiPSC-derived hematopoiesis, we blocked ER signals using ER antagonist 7α,β17-[9-[(4,4,5,5,5-Pentafluoropentyl)sulfinyl]nonyl]estra-1,3,5(10)-triene-3,17-diol (ICI 182,780) at 100 ng/ml (Tocris) in EB differentiation medium. Undifferentiated hiPSCs were transfected with ON-TARGETplusSMARTpool siRNA targeting ER-α (L-003401-00-0005, Dharmacon) for 24 h according to the manufacturer’s instructions. Non-targeting siRNA (D-001910-10-50, Dharmacon) was used as a negative control.

### Flow cytometry analysis

EBs were dissociated with Collagenase IV in 37 °C and 5 % CO_2_ for 2 h. Single cell suspension from dissociated EBs was resuspended in 3 % FBS-PBS. The cell were passed through a 70 μm cell strainer and incubated at 4 °C for 1 h with the following fluorochrome-conjugated mouse anti-human antibodies: CD31-PE, CD34-FITC, CD45-APC, CD235a-PE, and CD71-APC (all BD Biosciences) or their corresponding isotype controls. Anti-OCT4 (BD Biosciences) and anti-ER-α (Santa Cruz) staining was identified using Alexa 488- and 647-conjugated goat anti-mouse IgG (Invitrogen). After washing with 3 % FBS-PBS, the cells were stained with 7-amino actinomycin to exclude dead cells. Flow analysis was performed on a FACSCanto II running BD FACSDiva™ (BD Biosciences) and acquired data were analyzed using FlowJo version 10 (Tree Star, Inc.).

### Colony forming unit (CFU) assay

CFU assay was performed as previously described [32]. Briefly, 10,000 cells dissociated from EBs were plated into methylcellulose H4230 (STEMCELL Technologies) supplemented with 50 ng/ml SCF, 10 ng/ml GM-CSF, 10 ng/ml IL-3, and 3 units/ml erythropoietin. Hematopoietic cell clusters were counted on the basis of morphology after incubation for 10–14 days.

### Generation of hemangioblast and measurement of colony forming capability

Hemangioblasts were generated as previously described [19]. Briefly, 2 × 10^5^ hiPSCs were plated on Ultra-Low attachment plate in Stemline II medium (Sigma) containing BMP-4 (50 ng/ml) and VEGF (50 ng/ml) and incubated them for 48 h. Then, the half of the medium was replaced with the medium supplemented with BMP-4 (50 ng/ml), VEGF (50 ng/ml), SCF (20 ng/ml), thrombopoietin (20 ng/ml), and Flt-3L (20 ng/ml). After 3.5 days, the EBs were dissociated with 0.05 % trypsin–EDTA for 5 min and filtered through a 40 μm cell strainer. To expand hemangioblasts, the cells were resuspended in blast growth medium alone or supplementation of E2, propyl pyrazole triol (PPT, 100 nM) and diarylpropionitrile (DPN, 100 nM) and then incubated for 4–6 days. After 4–6 days, grape-like blast colonies were observed and counted in each culture condition. In order to assess colony-forming capability of blasts grown in each condition, blasts were resuspended in Stemline II medium and mixed with methylcellulose H4436 (STEMCELL Technologies). Then, cells were plated into a 12-well non-tissue culture plate. Hematopoietic cell clusters were counted on the basis of morphology after incubation for 15 days.

### Harvest of hematopoietic progenitors from human umbilical cord bloods (hUCBs)

Human UCB samples were obtained from normal full-term deliveries by Caesarian section at the Kangwon National University Hospital with the Kangwon National University Hospital Institutional Review Board (KNUH-IRB)-approved (IRB approved number: KNUH-2012-11-003-008) written consent from the patients. Mononuclear cells (MNCs) were isolated using Ficoll-Paque Plue (Pharmacia Biotech) and then resuspended in 1 % FBS-PBS. Hematopoietic progenitors expressing CD34+ antigen were enriched from MNCs by negative selection using StemSep isolation system (STEMCELL Technologies) according to the manufacturer’s instructions.

### Immunofluorescence staining

For immunofluorescence staining, undifferentiated hiPSCs and hemangioblast were fixed with 4 % paraformaldehyde for 10 min and then permeabilized with 0.5 % saponin in PBS. The cells were blocked with 10 % normal goat serum (Sigma) for 30 min at room temperature. The cells were then incubated with the following primary anti-human antibodies overnight at 4 °C: OCT4 (BD Biosciences), ER-α (Santa Cruz), and CD144 (BD Biosciences). The cells were washed twice with PBS and incubated with fluorochrome-conjugated secondary antibodies for 1 h at room temperature. The nuclei were counterstained with DAPI (Sigma) for 5 min. Fluorescent images were visualized with a fluorescence microscope (IX-71, Olympus).

### Data analysis

Values for all measurements are presented as mean ± SD. Comparisons for all experiments were performed with Student’s *t* test. Significance levels were set at *p* < 0.05.

## Additional files

10.1186/s13578-016-0111-9 Temporal change of ER-alpha during hiPSC-derived hematopoietic development.
10.1186/s13578-016-0111-9 Temporal changes (%) of ER-α and hematopoietic phenotypes during hiPSC-derived hematopoietic differentiation.
10.1186/s13578-016-0111-9 Optimization of E2 concentration for hematopoiesis.
10.1186/s13578-016-0111-9 siRNA-based Knockout of ER-alpha promotes hematopoietic differentiation.
