# Supplementary figures and images for: Improved hematopoietic differentiation of human pluripotent stem cells via estrogen receptor signaling pathway
Source: Cell Biosci. 2016 Aug 30;6(1):50. doi: 10.1186/s13578-016-0111-9 (PMC5006567; doi:10.1186/s13578-016-0111-9)

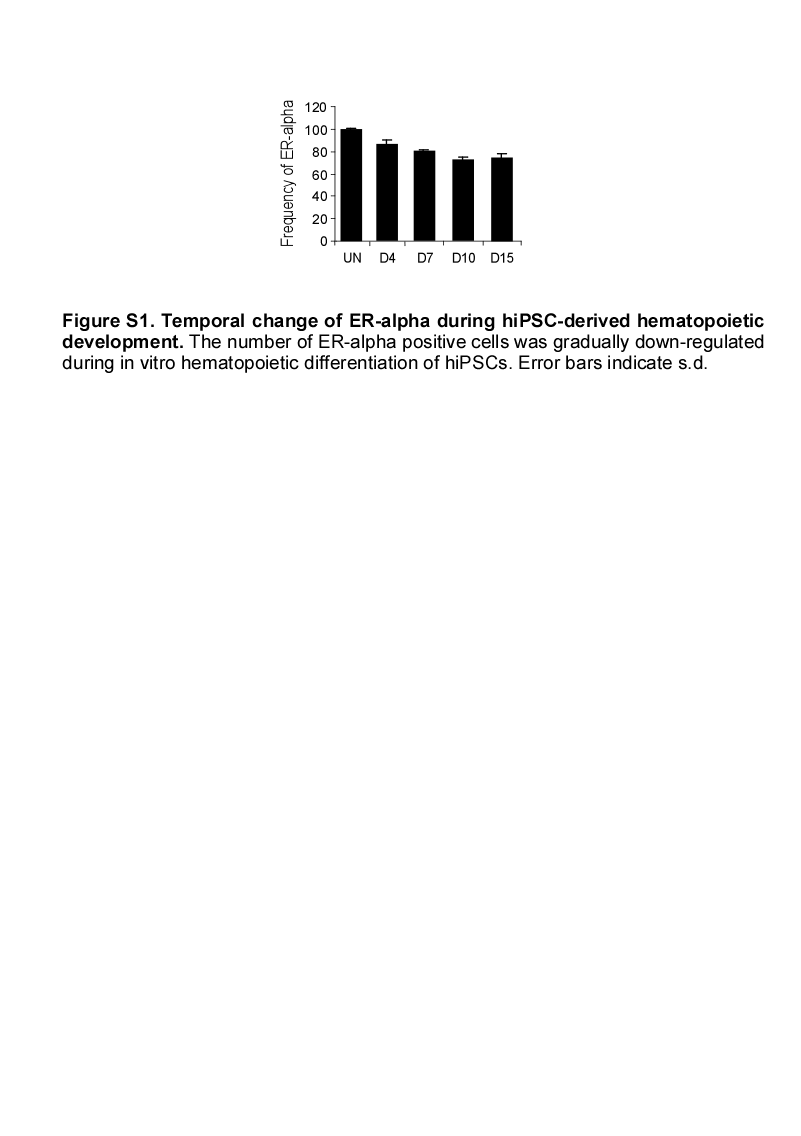

Supplement: Supplementary file 1 — 10.1186/s13578-016-0111-9 Temporal change of ER-alpha during hiPSC-derived hematopoietic development. [file 13578_2016_111_MOESM1_ESM.tif]

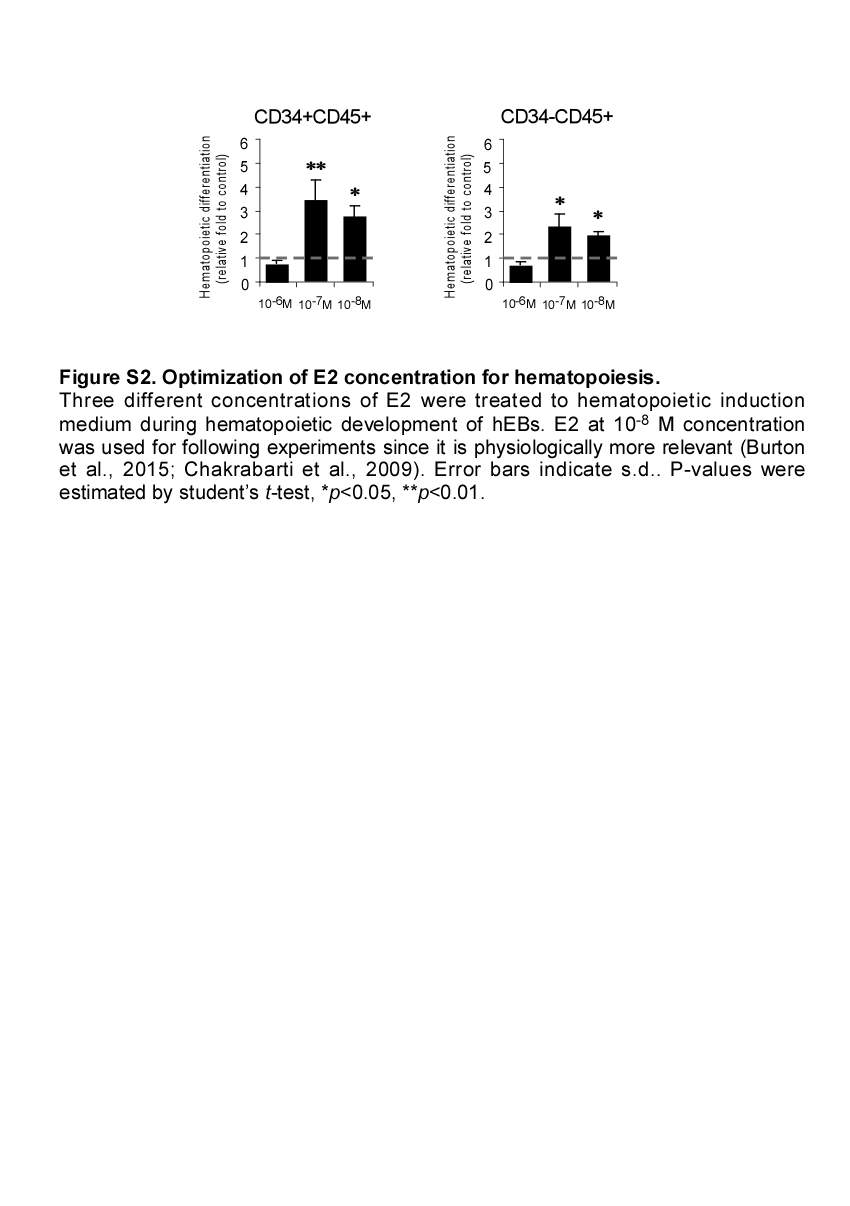

Supplement: Supplementary file 3 — 10.1186/s13578-016-0111-9 Optimization of E2 concentration for hematopoiesis. [file 13578_2016_111_MOESM3_ESM.tif]

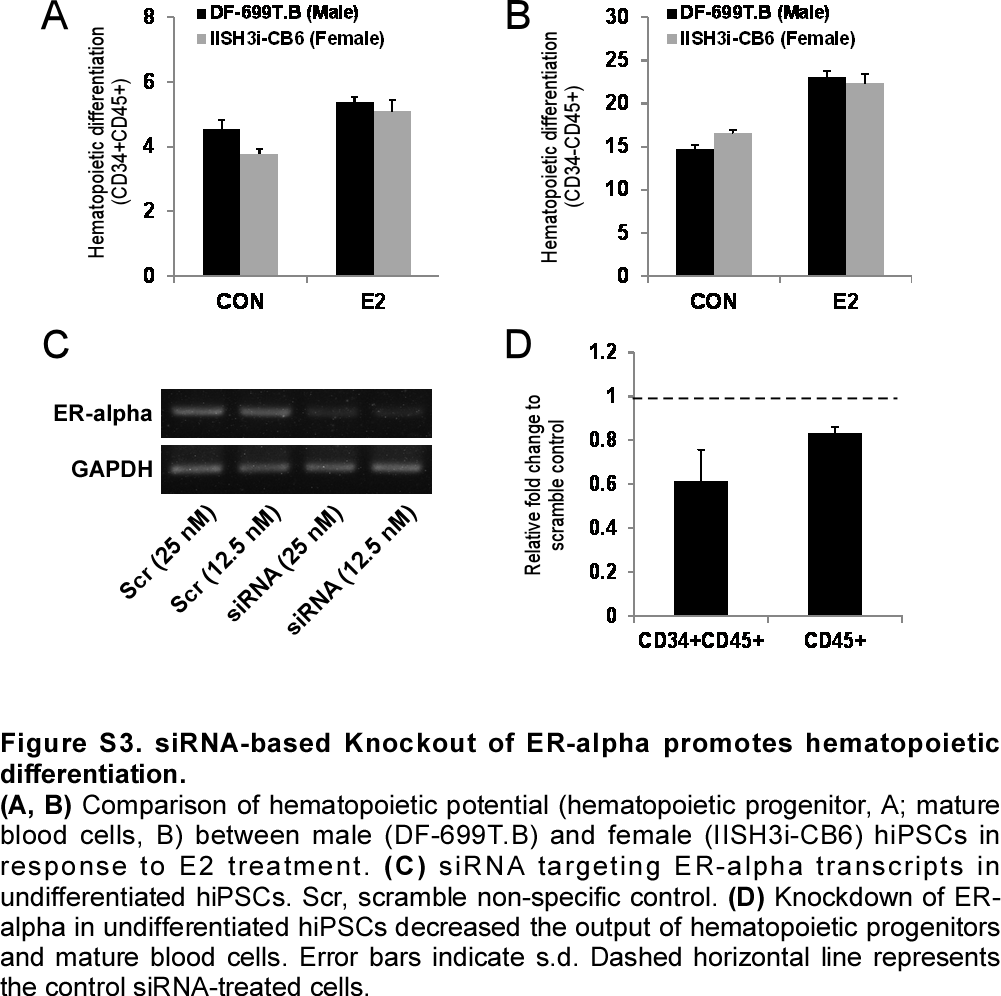

Supplement: Supplementary file 4 — 10.1186/s13578-016-0111-9 siRNA-based Knockout of ER-alpha promotes hematopoietic differentiation. [file 13578_2016_111_MOESM4_ESM.tif]
