# Supplementary material for: Improved hematopoietic differentiation of human pluripotent stem cells via estrogen receptor signaling pathway
Source: Cell Biosci. 2016 Aug 30;6(1):50. doi: 10.1186/s13578-016-0111-9 (PMC5006567; doi:10.1186/s13578-016-0111-9)
Supplement: Supplementary file 2 — 10.1186/s13578-016-0111-9 Temporal changes (%) of ER-α and hematopoietic phenotypes during hiPSC-derived hematopoietic differentiation. [file 13578_2016_111_MOESM2_ESM.docx]

**Table S1.** Temporal changes (%) of ER-α and hematopoietic phenotypes during hiPSC-derived hematopoietic differentiation.

| **D4** | | **D7** | | **D10** | | **D15** | |
| --- | --- | --- | --- | --- | --- | --- | --- |
| ER-α^+^CD31^+^ | ER-α^-^CD31^+^ | ER-α^+^CD31^+^ | ER-α^-^CD31^+^ | ER-α^+^CD31^+^ | ER-α^-^CD31^+^ | ER-α^+^CD31^+^ | ER-α^-^CD31^+^ |
| 0.38±0.19 | 0.02±0.001 | 1.48±0.13 | 0.20±0.021 | 4.63±1.51 | 0.74±0.003 | 8.94±2.37 | 0.98±0.473 |
| ER-α^+^CD34^+^ | ER-α^-^CD34^+^ | ER-α^+^CD34^+^ | ER-α^-^CD34^+^ | ER-α^+^CD34^+^ | ER-α^-^CD34^+^ | ER-α^+^CD34^+^ | ER-α^-^CD34^+^ |
| 0.32±0.213 | ND | 3.30±0.28 | 0.13±0.031 | 5.65±1.23 | 0.92±0.098 | 7.96±1.72 | 0.38±0.091 |
| ER-α^+^CD45^+^ | ER-α^-^CD45^+^ | ER-α^+^CD45^+^ | ER-α^-^CD45^+^ | ER-α^+^CD45^+^ | ER-α^-^CD45^+^ | ER-α^+^CD45^+^ | ER-α^-^CD45^+^ |
| 0.67±0.42 | 0.09±0.067 | 1.62±0.04 | 0.03±0.004 | 1.78±0.06 | 0.17±0.072 | 5.89±1.31 | 0.19±0.082 |
